# Supplementary figures and images for: Naphthoquinone preferentially pairs with non-proton-pumping NADH dehydrogenase for respiratory electron transport
Source: PLoS Genet. 2025 Sep 24;21(9):e1011877. doi: 10.1371/journal.pgen.1011877 (PMC12510636; doi:10.1371/journal.pgen.1011877)

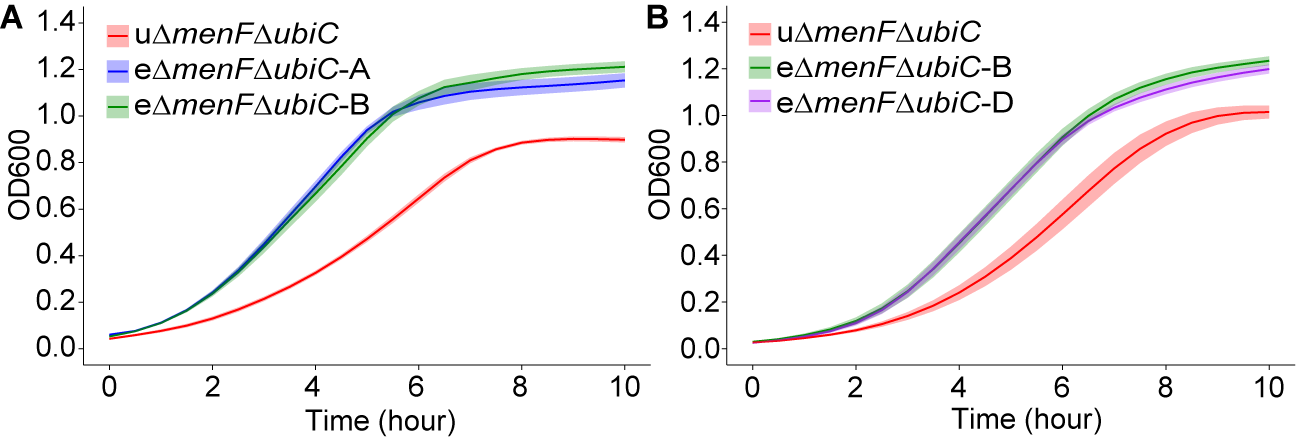

Supplement: S1 Fig — (A) The lineage B has a point mutation in ubiE but grows similar to lineage A that has wild type ubiE sequence. (B) The lineage B has a point mutation in pykF but grows similar to lineage D that has wild type pykF sequence. Values represent means from four biological replicates (each with three technical replicates); shaded bands indicate standard error of the mean. (TIF) [file pgen.1011877.s001.tif]
