# Supplementary material for: Naphthoquinone preferentially pairs with non-proton-pumping NADH dehydrogenase for respiratory electron transport
Source: PLoS Genet. 2025 Sep 24;21(9):e1011877. doi: 10.1371/journal.pgen.1011877 (PMC12510636; doi:10.1371/journal.pgen.1011877)
Supplement: S1 Table — (PDF) [file pgen.1011877.s002.pdf]

S1 Table: List of mutated genes in strains isolated at the endpoint of the evolutionary trajectory

**Strain**	**Mutations**		
	**Mutations enabling optimization to the growth medium**	**Common mutations across all lineages**	**Mutations not common across all lineages**
eΔ*menF*Δ*ubiC*-A	Δ82 bp in *pyrE-rph* intergenic region at genomic location 3815859	PdhR Box: C→T at genomic location 1165961	*pykF*: G→T at genomic location 1756265
eΔ*menF*Δ*ubiC*-B	Δ82 bp in *pyrE-rph* intergenic region at genomic location 3815859	*pdhR*: G→T at genomic location 122095	*pykF*: A→C at genomic location 1756920, *ubiE*: G→A at genomic location 4019576
eΔ*menF*Δ*ubiC*-C	*rpoC*: GAC→TAC at genomic location 4187213	PdhR Box: G→A at genomic location 1165953	
eΔ*menF*Δ*ubiC*-D	Δ82 bp in *pyrE-rph* intergenic region at genomic location 3815859, *rpoC*: CGT→CTT at genomic location 4188573	PdhR Box: A→T at genomic location 1165956	*ubiE*: C→T at genomic location 4019155
