# Supplementary material for: Naphthoquinone preferentially pairs with non-proton-pumping NADH dehydrogenase for respiratory electron transport
Source: PLoS Genet. 2025 Sep 24;21(9):e1011877. doi: 10.1371/journal.pgen.1011877 (PMC12510636; doi:10.1371/journal.pgen.1011877)
Supplement: S2 Table — (PDF) [file pgen.1011877.s003.pdf]

S2 Table: The table summarizes the representative genes associated with specific respiratory quinone biosynthesis pathways and NADH dehydrogenases and their custom e-value cutoffs

	**Gene name**	**HMM hits e-value cutoff**
Classical NQ biosynthesis	*menB*	4.27E-27
	menC	2.09E-21
	menF	5.95E-63
Futalosine NQ biosynthesis	*mqnA*	3.20E-34
	mqnC	4.25E-128
	mqnD	2.28E-45
UQ biosynthesis	*ubiA*	5.98E-25
	ubiC	5.42E-11
	ubiG	7.19E-04
NDH-1	*nuoA*	1.93E-01
	nuoE	1.12E-03
	nuoJ	6.91E-02
NDH-2	*ndh*	7.83E-16
