# Supplementary material for: Naphthoquinone preferentially pairs with non-proton-pumping NADH dehydrogenase for respiratory electron transport
Source: PLoS Genet. 2025 Sep 24;21(9):e1011877. doi: 10.1371/journal.pgen.1011877 (PMC12510636; doi:10.1371/journal.pgen.1011877)
Supplement: Attachment — Submitted filename: Response to Reviewers.pdf [file pgen.1011877.s007.pdf]

September 9, 2025

Manuscript No.: PGENETICS-D-25-00915

****

Reviewers' Comments: **Black Text**

Responses by authors: **Blue Text**

Line numbers mentioned in this document refer to revised manuscript

****

We are pleased that the reviewers unanimously recognized the value of our work. We have carefully addressed all remarks by the reviewers.

****

Reviewer #1

The manuscript by Khairnar et al. describes an in-vitro evolution study on the strategies of *E.coli* strains deleted in both respiratory quinones (low-potential naphthoquinones and high-potential menaquinones) to suppress the challenges generated by the compromised respiratory chain. Naphthoquinone-deletion was found to be leaky due to the presence of an iso-chorismate synthase. Strains were found to recover increased rates of respiration via routing electrons from NADH through the non-proton-pumping NDH-2 enzyme rather than through Complex I. The experiments are exhaustive and well-controlled and the results are presented in a concise but complete manner. The obtained conclusions are extremely pertinent to our understanding of the thermodynamic constraints guiding evolution of energy converting electron transfer chains. I therefore recommend publication of this manuscript in PloS Genetics.

Thank you for the appreciation of our work.

I have only two minor comments:

1) Line 71: "… ndh (gene encoding…" must read "… ndh gene (encoding …"

   Modified as suggested (line no. 93).

2) Lines 120-129. The authors mainly attribute the reason for favoring NDH-2 over Complex I to the higher catalytic efficiency of the former thereby suppressing ROS generated by semiquinone radicals. There are two things to take into account in this context. (a) NDH-2 performs a 2-electron reduction from NADH to quinone via its highly cooperative flavin cofactor while the reduction from the iron-sulfur-center-chain to quinone in Complex I necessarily has to go one-by-one. This implies that basically no semiquinone radical is produced by NDH-2 while Complex I has been shown by a host of experiments to be one of the two major sources together with the Qo-site of Complex III) of ROS in respiratory chains. (b) The driving deltaG from NADH to naphthoquinones is much lower than that to benzoquinones. Since Complex I does additional work

(pumping protons and generating a pmf), its function becomes quickly compromised in the presence of the produced pmf. It therefore will stall (the reaction NADH –> quinone + pmf will go to equilibrium) and no electrons will reach the downstream ETS enzymes. This problem is much less pronounced for the benzoquinones due to the increase in deltaG resulting from their higher redox potential. A short mentioning of these facts in this paragraph might be warranted.

We greatly appreciate this remark. We have suitably integrated this information in the revised manuscript (line no. 135-145). Given the importance of this suggestion, we have also acknowledged this help in the acknowledgement section.

****

Reviewer #2

This manuscript investigates the metabolic adaptation of naphthoquinone (NQ)–dependent *Escherichia coli*, with a particular focus on the electron transport chain (ETS). The authors performed adaptive laboratory evolution (ALE) of *E. coli* strains lacking menF and ubiC, two genes essential for respiratory quinone biosynthesis. They subsequently analyzed mutations in evolved clones and identified upregulation of ndh, encoding the non-proton-pumping NADH dehydrogenase, suggesting its preferential pairing with NQ. Overall, the study is well-executed, and the results are clear and compelling. However, several issues, particularly related to writing style and clarity, need to be addressed before publication. Once these concerns are resolved, I would be happy to recommend the manuscript for publication in PLOS Genetics.

Thank you for an encouraging review of our work.

Major Comments

1) Clarity and contextualization of prior work: the text is sometimes overly abstract, particularly when introducing previous studies. Please provide more detailed explanations so that readers can follow without relying heavily on cited literature. For example:

    (a) Line 39: Clarify the specific observation with evolved NQ-dependent *E. coli*. The sentence “The growth improvement of the NQ-dependent strain imparted by the loss of function of PdhR creates a mechanistic paradox” is vague. Please state what the actual observation was.

    Modified as suggested (line no. 58-63).

    (b) Line 43: Explain the mechanistic relationship between PdhR and NDH-2. Also clarify what “this study” refers to in this context.

    Modified as suggested (line no. 58-59).

(c) Line 46: Revise to explicitly state the roles of ubiC and menF in NQ and UQ biosynthesis, and confirm that ubiquinones (UQs) are the canonical respiratory quinones.

Modified as suggested (line no. 51-52 and 69-70).

(d) Line 59: Clearly indicate that "u" and "e" before strain genotypes designate "unevolved" and "evolved," respectively.

Modified as suggested (line no. 89-90).

(e) Abstract: The sentence "Using adaptive laboratory evolution of a naphthoquinone-dependent Escherichia coli strain, we previously showed the fitness advantage of high-potential quinones" requires elaboration. Please explain what is meant by the energy potential of quinones and why this distinction matters.

Modified as suggested line no. (16-17).

(f) Line ~120: Expand the description of the custom hidden Markov Model profiles: what genes were targeted, what metabolites/enzymes they correspond to, and why these models were developed.

We have suitably modified this section (line no. 158-161). We recently performed a large-scale search for respiratory Complex I across the entire prokaryotic genome space (Shirstah *et al.*, Genome Biology and Evolution, 2025). While working on this project we compared various search methods and found HMM as the most unbiased and powerful approach. We have cited the paper for readers' benefit.

2) Figure 2 and evolved lineages: the authors obtained four independently evolved strains, but only the lineage A isolate was studied in depth. Were additional mutations (beyond the ndh upstream region) identified in the other lineages? Please elaborate on whether parallel or distinct adaptive mechanisms were observed.

We always perform our adaptive laboratory evolution experiments with three or more independent lineages. This approach allows us to filter hitchhiker mutations and find mutations that are likely to be causal for the improved phenotype.

While three evolved lineages fixed mutations in the PdhR box region of the *ndh* gene, the fourth lineages showed mutation in the PdhR gene itself. As reported in the manuscript, this lineage also acquired the same adaptive strategy of selectively upregulating *ndh* expression.

Further, we have added the following paragraph (line no. 119-127) to provide details of genetic changes: "*Additionally, all evolved lineages fixed well-characterized mutations in RNA polymerase subunit or in the intergenic region between pyrE and rph (Supp. Table 1). These mutations provide a growth advantage on the media used during the adaptive laboratory evolution [12]. While there was no other common mutation observed in the evolved strains, two notable mutations were in pyruvate kinase (pykF) and methyltransferase (ubiE). Given these mutations were present in more than one evolved lineage, we performed growth comparison between strains with and without these mutations. While we can not completely rule out any additional benefit provided by these mutations, we did not observe any growth difference between strains with and without these genetic changes.*"

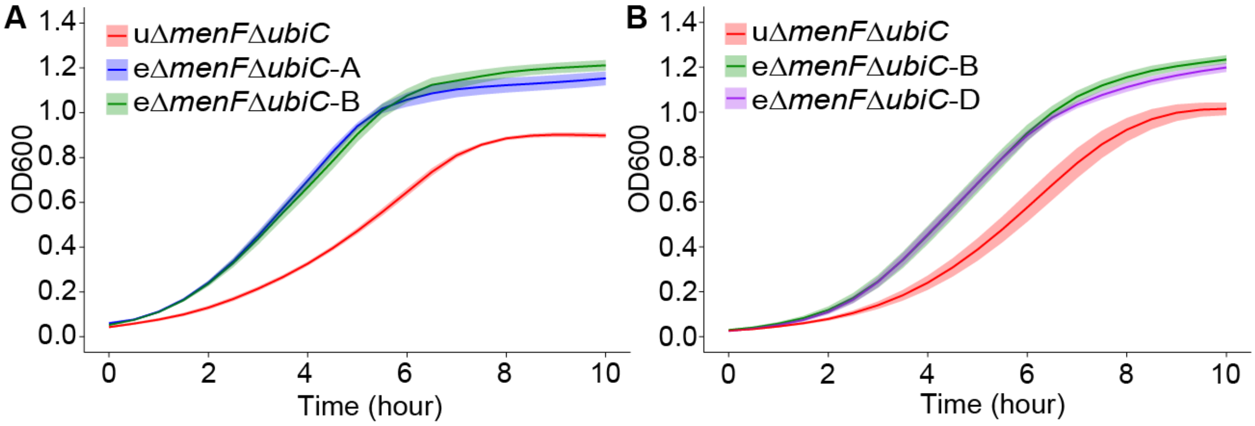

Supp. Figure 1: Growth profile of Δ*menF*Δ*ubiC strains* to assess influence of additional mutations observed during adaptive evolution. (A) The lineage B has a point mutation in *ubiE* but grows similar to lineage A that has wild type *ubiE* sequence. (B) The lineage B has a point mutation in *pykF* but grows similar to lineage D that has wild type *pykF* sequence. Values represent means from four biological replicates (each with three technical replicates); shaded bands indicate standard error of the mean.

3) Overview of ETS and quinone coupling: for clarity, please expand on how quinones interact with the electron transport chain. A concise overview figure or schematic showing the coupling of NQ and UQ with different dehydrogenases and terminal oxidases would strengthen the manuscript.

We have added a schematic to show the *E. coli* aerobic ETS.

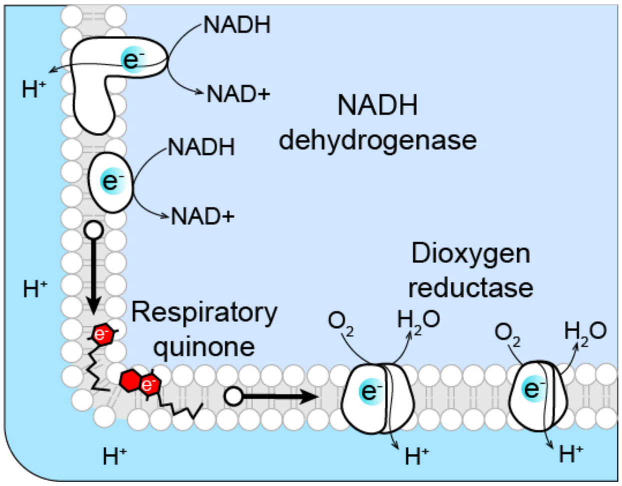

Figure 1A: A simplistic scheme for the *E. coli* aerobic ETS working between NADH and oxygen. Electrons donated by NADH travel to dioxygen reductase mediated by respiratory quinones and finally accepted by oxygen.

****
